# Improving VMAT dose calculation accuracy and planning quality via a GPU‐accelerated Fourier transform dose calculation algorithm

**DOI:** 10.1002/acm2.70002

**Published:** 2025-02-07

**Authors:** Kenny Guida, Chaoqiong Ma, Joy Patel, Krishna Reddy, H. Harold Li

**Affiliations:** ^1^ Department of Radiation Oncology University of Kansas Cancer Center Kansas City Kansas USA

**Keywords:** dose calculation, FTDC, GPU, lung SBRT, treatment planning, VMAT

## Abstract

**Background:**

Inverse planning typically utilizes fast, less accurate dose calculation algorithms *during* the iterative optimization process, thus leading to dose calculation errors (DCEs) and suboptimal plans that often require dose normalization and/or plan re‐optimization.

**Purpose:**

A graphic processing unit (GPU) accelerated Fourier transform dose calculation (FTDC) was recently commissioned at our institution during the Eclipse treatment planning system (Varian Medical Systems) v18.0 upgrade. We hypothesize that FTDC could reduce DCEs and planning failure rates (PFRs) compared to its predecessor, multi‐resolution dose calculation (MRDC), while improving efficiency through utilization of GPUs.

**Methods:**

Fifty lung SBRT plans were optimized with MRDC and FTDC dose calculation algorithms. Acuros XB (AXB) was then used for final dose calculations. DCEs for target and organ‐at‐risk (OAR) were calculated as the percent difference between AXB and dose calculated at the final optimization step. Plan quality was assessed using an in‐house planning scorecard where PFRs were calculated as the percentage of plans that had a plan score less than 90% with optimal plans scored at 100%.

**Results:**

FTDC showed excellent agreement with AXB in terms of planning target volume (PTV) coverage, as PTV D95% DCE_FTDC_ averaged 0.8% ± 0.9%, compared to DCE_MRDC_’s −2.5% ± 3.2%. DCEs for thoracic OARs were reduced with less variation when optimizing with FTDC as compared to MRDC. FTDC had a PFR of 10% (5 out of 50) versus MRDC's 32% (16 out of 50). The subsequent re‐optimization rate resulted from a plan normalization of 3% or greater was 4% for FTDC compared to MRDC's 38%. FTDC with GPU acceleration reduced optimization time by 75% on average compared to MRDC without GPU acceleration.

**Conclusions:**

FTDC shows more accurate dose calculation accuracy compared to MRDC. Its use during the optimization process improved planning quality and efficiency assisted with GPUs.

## 1 INTRODUCTION

In radiation therapy, dose calculation accuracy in treatment planning systems (TPSs) plays a pivotal role in assisting decision‐making during the planning process that will impact patient treatment.^1^, ^2^, ^3^, ^4^ Over the past three decades, final dose calculation has improved greatly from correction‐based methods to convolution/superposition algorithms and Boltzmann transport equation solvers, such as Acuros XB (AXB).^1^, ^5^ With the evolution of dose calculation algorithms like AXB, clinical dose calculations are approaching the accuracy of Monte Carlo, long considered the gold standard in dose calculation.^6^ However, dose calculation *during* the inverse optimization process has often relied on quicker and less accurate algorithms, a tradeoff that is made due to the extensive computational power and time required during the optimization process, which leads to disparities seen in final dose calculation, that is, dose calculation errors (DCEs).^7^, ^8^ As a result, dose normalization and/or plan re‐optimization are often required to meet the original planning goals, thus reducing planning efficiency. It is possible to accept suboptimal plans due to limited planning time for each patient in a busy clinic. Furthermore, both Dogan and Jeraj suggest that the use of the most accurate dose‐calculation algorithm during inverse planning processes may even minimize the chance of guiding the optimization to some suboptimal solutions, thus reducing optimization convergence errors.^9^, ^10^

Intensity modulated radiation therapy (IMRT) dose calculation and optimization processes have been improved incrementally over the past decade. Currently, volumetric modulated radiation therapy (VMAT) is the most dominant IMRT delivery technique, as it possesses utilitarian capabilities of delivering quality treatment plans for many disease sites and for both conventional and hypofractionated treatment regimens. With Eclipse TPS v13.5 (Varian Medical Systems, Palo Alto, CA), the photon optimizer (PO) algorithm was enabled for VMAT optimization and led to reduced plan complexity and increased agreement between calculated and measured dose, as compared to its predecessor, the progressive resolution optimizer (PRO).^11^, ^12^, ^13^ PO defines the structures, dose volume histogram (DVH) calculations, and dose sampling with a single matrix for the image set and enables users to select from voxel resolutions of 1.25 and 2.5 mm. In PO, the optimizer utilizes four multiresolution levels that progressively increase the total number of dose calculation segments. Dose calculation within the optimizer has been performed with the multi‐resolution dose calculation (MRDC) algorithm. MRDC is a pencil beam dose calculation algorithm based on convolution/superposition principles that is used for fast dose calculation within PO during plan optimization. This algorithm employs 3D convolution scatter computation with point spread functions modeled with Monte Carlo.^14^ While MRDC provides fast dose calculation at each progressive step and level during optimization, in large part due to a simplified pencil beam dose calculation approach, it is less accurate than photon dose calculation algorithms used for final dose calculation, including Acuros XB (AXB) and Analytical Anisotropic Algorithm (AAA).^15^ Previous studies have shown discrepancies between planning target volume (PTV) coverage estimated by MRDC, as final dose calculation with AAA showed a 5%–10% drop in PTV dose.^7^, ^15^ In many instances, planners adjust the PTV dose objectives to higher levels through plan normalization to achieve tumor coverage due to disparities seen between MRDC and AAA; situations with large plan normalization of 3% or greater may necessitate additional plan optimization.^7^ A further improvement in dose calculation algorithms within the optimizer is desirable, which could increase planning accuracy and efficiency and potentially lead to better treatment plans.

With the arrival of Eclipse v18, the TPS provides users the ability to utilize a new dose calculation algorithm, Fourier transform dose calculation (FTDC), within PO. FTDC is a convolution/superposition dose calculation algorithm that can be enabled for VMAT dose calculation within the optimizer. This new algorithm relies on convolution kernels based on Monte Carlo‐generated point spread functions, which are then superimposed based on the energy spectrum before being convolved with a spectrally attenuated fluence via fast Fourier transform. Similar to MRDC, FTDC models extra‐focal photons as secondary sources, as well as electron contamination. To enhance the computation time and optimization efficiency, graphic processing units (GPUs) can be utilized when enabling FTDC for dose calculation during VMAT optimization. FTDC is the sum of primary, secondary, and electron contamination sources. The primary fluence grid is modeled after AXB and AAA but uses simpler models for the three aforementioned components to improve dose calculation speed during plan optimization.^14^ To improve dose computation in heterogeneous media, FTDC utilizes a TERMA scaling method based on beam size and density. A recent study from Laakkonen et al. showed that FTDC compares favorably to both Monte Carlo and AXB and is more accurate than AAA for small fields passing through lung tissue in phantom studies.^16^ Laakkonen et al. presented depth dose curves through water‐air‐water and water‐cork‐water geometries for AAA, AXB, FTDC, and Monte Carlo for 6 MV beams with small field geometries, as seen in Figure 4 in their study. The depth dose curves highlighted the improvements made in the FTDC algorithm for small field geometries (10 mm × 10 mm), as the new FTDC algorithm closely matches AXB and Monte Carlo dose calculations; the previous FTDC algorithm and AAA tended to overestimate dose in both air and cork. In addition, Laakonen et al. highlighted the application of FTDC in a lung patient test case, showing that FTDC agreed well with AXB, with a gamma pass rate of 99.8% using 2%/2 mm criteria.^16^ Dose differences seen between optimizer and final dose calculations have been an issue with IMRT and VMAT, especially for plans involving small field geometries in the lungs, where loss of lateral electronic equilibrium can occur.^17^ Jeraj et al. noted that systematic errors that arise from pencil beam dose calculation algorithms can affect both the final dose calculation and optimization convergence errors. In their work, systematic and convergence errors in lung IMRT cases increased when switching from superposition to pencil beam dose calculation algorithms.^10^

With discrepancies seen between final and optimization dose calculation algorithms for cases involving small field sizes and heterogeneous media in previous iterations of Eclipse TPS, we decided to investigate the accuracy of FTDC for lung SBRT plans. Coupled with the fact that SBRT plans are hypofractionated and deliver over 8 Gy per fraction, dose calculation accuracy is especially critical. In this study, the accuracy and efficiency of FTDC (v18.0) and MRDC (v15.6) algorithms were examined for a complement of 50 lung SBRT cases, spanning different regions of the lungs (central, island, and peripheral) and consisting of a variety of PTV volumes. Dose calculation accuracy for PTV and organ‐at‐risk (OAR) volumes was assessed for each algorithm and compared to final dose calculation performed with AXB v18.0. Plan quality was assessed for plans optimized with both FTDC and MRDC algorithms using dosimetric scorecards. It is important to assess improvements in planning efficiency, as clinicians require streamlined processes to navigate ever‐increasingly busy environments. Gains in efficiency during plan optimization, through improved agreement between optimized and final dose calculations, as well as gains in speed, could improve patient throughput.

## 2 METHODS

### 2.1 Patient cohort and planning methodology

A retrospective analysis of 50 previously treated lung SBRT cases was conducted for this study. Two plans were generated for each of the patients utilizing available dose calculation algorithms within PO: MRDC and FTDC. All cases were planned to deliver 50 Gy in five fractions to the PTV. A breakdown of the patient cases is found in Table 1.

**TABLE 1:** A breakdown of the cases included in this study.

Lung SBRT cases data, including location, volume, and planning information	
Lung SBRT case data	
Location	
Central	13
Island	15
Peripheral	22
PTV Volume (cc)	
Minimum	4.7
Maximum	68.7
Mean	22 ± 16
Treatment Planning Data	
KBP Optimization	31
Manual Optimization	19
PO Standard Resolution (2.5 mm)	33
PO High Resolution (1.25 mm)	17
Standard MLC (5 mm)	17
High‐Definition MLC (2.5 mm)	33

*Note*: Location and PTV size varied across the patient cohort, as well as optimization techniques and MLC types.

All plans employed 6X‐FFF for beam energy and were delivered on either a Varian TrueBeam (standard MLC) or Edge (high definition MLC (HD‐MLC)). In keeping the optimization consistent between all plans, the original beam geometry and optimization parameters were utilized for each case; typical beam geometry utilized 2–3 partial VMAT arcs, with plan designs using a mix of coplanar and non‐coplanar (± 10 degree) table arrangements. Plans were optimized to achieve a PTV D95% of 5000 cGy in five fractions and achieve normal tissue tolerances for nearby OARs; clinical goals and tolerances applied to these plans are listed in Table 2. The original lung SBRT plans were devised by a multitude of dosimetrists with varied levels of experience and differences in optimization strategies; some planners relied on an in‐house knowledge‐based planning (KBP) model for optimization, and some planners enabled high resolution (1.25 mm grid) for dose calculation during plan optimization. Keeping the original optimizations allowed us to further investigate the effects of structure resolution and KBP modeling on SBRT plan optimization and calculation. Convergence mode was enabled to force the optimizer to converge at each resolution level within PO. Intermediate dose calculation was performed after MR Level 4 in the optimization. For plans using FTDC, GPU calculation was enabled in PO; GPU calculation is not available for MRDC in the optimizer. PO v18 (PO18) was used for FTDC plans, while PO v15 (PO15) was employed for MRDC plans. Final dose calculation was performed with AXB v18 using a 1.25 mm dose grid.

**TABLE 2:** Lung SBRT clinical goals and constraints.

Lung SBRT Clinical Goals and OAR Tolerances	
Target	Goal
PTV_5000	D95% ≥ 5000 cGy
	D99% ≥ 4625 cGy
OAR	Constraint
Chestwall	D30cc < 3000 cGy
Esophagus	D5cc < 1950 cGy
	D0.03cc < 3500 cGy
Great Vessels	D10cc < 4700 cGy
	D0.03cc < 5300 cGy
Heart	D15cc < 3200 cGy
	D0.03cc < 3800 cGy
Lung‐GTV	V1350cGy < 37%
Ribs	D5cc < 4500 cGy
	D0.03cc < 5700 cGy
SpinalCord	D1.2cc < 1560 cGy
	D0.35cc < 2200 cGy
	D0.03cc < 2800 cGy
Trachea	D5cc < 3200 cGy
	D0.03cc < 4000 cGy

### 2.2 Dose calculation error

To assess potential improvements in plan optimization through FTDC, it is important to assess DCEs and optimization‐convergence errors (OCEs) that can be propagated through plan optimization. In previous works, DCEs and OCEs were identified in plans that used less accurate methods, such as convolution/superposition algorithms, for dose calculation in the optimizer and final dose calculation.^9^, ^10^ Convergence errors tend to be a consequence of systemic errors, such as DCEs.^10^ Using a more sophisticated dose calculation, Monte Carlo (MC), for final dose calculation, and eventually even *during* optimization, allowed Dogan et al. to identify DCEs and OCEs for targets and OARs.^9^ In their work, Dogan et al. planned 10 IMRT head and neck cases, using convolution/superposition algorithms during optimization and final dose calculation (plan A); plans were recalculated with MC for final dose calculation (plan B). They calculated dose prediction error (DPE) as the percent difference between DVH metrics yielded between plans B and A, under the assumption that MC was the more accurate algorithm.

In our work, the concept remains similar, with the optimization calculations being set to MRDC and FTDC, with the final dose calculation fixed to AXB, the most accurate commercial final dose algorithm available on the Eclipse platform and comparable with MC.^6^ To determine DCE for both FTDC and MRDC, we compare the dose metrics, *x*, determined at the final step in optimization, *D_opt_*, and after final dose calculation, *D_AXB_*; therefore, the DCE for each structure and PO dose calculation algorithm (DCE_FTDC_ and DCE_MRDC_) is given as:

(1) $$DCE_{PO,x}\left(\%\right)=\frac{\left(D_{x}^{AXB}-D_{x}^{Opt}\right)}{D_{x}^{AXB}}.$$

Dosimetric data was also broken down into tumor location to determine if surrounding tissue density affected DCE. Tumor location was broken down into three regions: peripheral, island, and central. DCE_FTDC_ and DCE_MRDC_ were assessed for PTV and OAR metrics for each tumor location. A case study was performed to further dive into the impact of tumor location on DCE.

Paired T‐Tests were performed, comparing DCE_FTDC_ versus DCE_MRDC_ for each dose metric analyzed in this study. In comparing optimization techniques, MLC model, and dose grid resolution, paired T‐Tests were utilized to evaluate DCE_FTDC_ and DCE_MRDC_ for PTV D95% and D99%.

### 2.3 Planning failure rates

To quantitatively analyze plans optimized with MRDC and FTDC, an Eclipse Scripting Application Programming Interface (ESAPI) tool called PlanScoreCard was utilized to score both original and normalized/reoptimized plans; PlanScoreCard is available for free on the Varian Medical Affairs GitHub.^17^, ^18^ Dosimetric scorecards utilize multiple piecewise linear scoring functions to assess plans against established quality metrics.^17^, ^19^ We created a dosimetric scorecard based on our department standards and provided an example in Appendix A. The dosimetric scorecard was loaded by the ESAPI tool and used to analyze plans, providing composite scores based on DVH criteria for PTVs and all OARs. The composite scores were used to quantify plan quality, similar to other commercial software utilized in clinics and global plan challenges.^19^, ^20^ The optimal plans were scored at 100%, and planning failure rates (PFRs) were calculated as the percentage of plans that had a plan score less than 90%. Clinical plans with suboptimal scores would likely be re‐optimized to improve plan quality.

To assess plan complexity, duty cycle and plan complexity scores were captured for each plan in the study. Duty cycle was determined by the total plan MU divided by the dose per fraction, in cGy. The plan complexity metric utilized in this work is based off the work of Younge^21^; the plan complexity score, *M*, is defined as

(2) $$M=\frac{1}{MU}\;\sum_{i=1}^{N}MU_{i}\;\times \frac{y_{i}}{A_{i}},$$

where *M* is equal to the sum of all control point apertures from *i* = 1 to N, *MU* is the total plan MU, MU*_i_* is the MU per aperture *i*, *A_i_* is the aperture area for a specified aperture, and *y_i_* is the aperture perimeter.

### 2.4 Optimization efficiency

With the advent of FTDC in dose calculation during plan optimization, it is important to understand its impact on efficiency. To quantify optimization efficiency, optimization duration was timed from MR1 through MR4 for all plans in this study. Our treatment planning service has noted that optimization times can be affected by dose grid resolution used in plan optimization, as PO provides the user with two options: normal (2.5 mm) and fine (1.25 mm). With our clinical plans using both settings, we further split our optimization efficiency investigation into these two categories of PO dose grid sizes. Another setting that we believe can influence optimization efficiency is the use of KBP models. Optimization times were again separated based on the use of manual optimization and our in‐house lung SBRT KBP Model.

## 3 RESULTS

### 3.1 Dose calculation error

**FIGURE 1:**
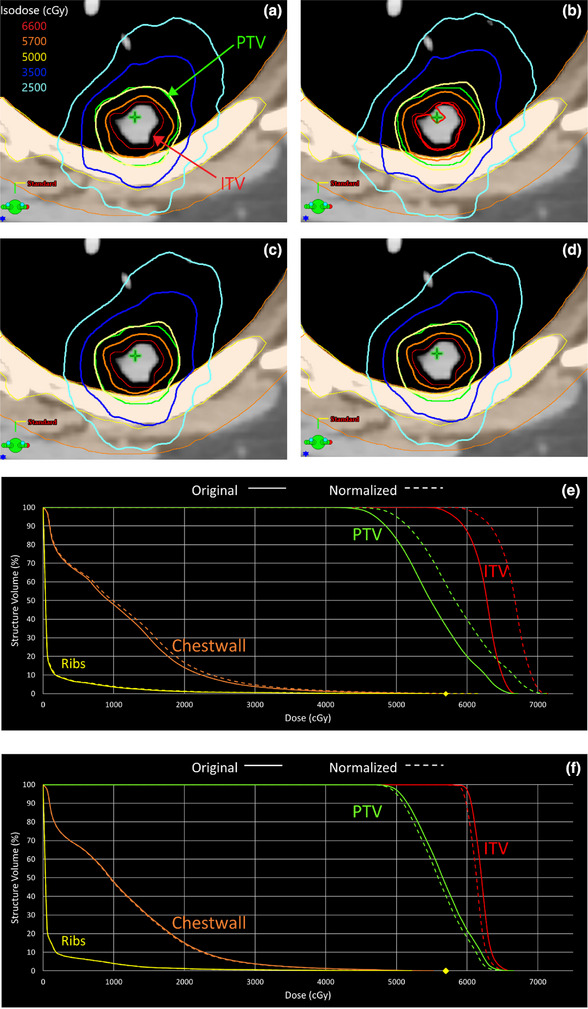
(a)–(f) Axial slices of MRDC plans (a—original, b—normalized up 6%) and FTDC plans (c—original, d—normalized down 1.2%) for a peripheral lung tumor. The DVHs provided show the MRDC (e) and FTDC (f) plan comparisons for PTV and ribs. Solid line: original; dashed: normalized. A yellow diamond marks the D0.03cc < 5700cGy constraint used for ribs; as seen in (e), normalizing the MRDC plan to achieve coverage causes the rib dose to exceed the constraint. DVH, dose volume histogram; FTDC, Fourier transform dose calculation; MRDC, multi‐resolution dose calculation.

Figure 1a–f shows a peripheral lung case that is overlapping the ribs. In this case, the MRDC plan (1a) yielded a PTV D95% of 4700 cGy and a D99% of 4470 cGy, while the FTDC plan (1c) achieved 5060 and 4906 cGy, respectively. Upon plan normalization, the MRDC plan (1b, e) required nearly 6% normalization to achieve the desired coverage, while also causing the Ribs D0.03cc to increase from 5635 to 5992 cGy, failing our constraint. In contrast, the normalized FTDC plan (1d, f) only required a normalization of 1.2% downward to achieve D95% of 5000 cGy. Using the scorecard to assess plan quality, the MRDC plan (1a) only scored 87.7%, meanwhile the FTDC plan (1b) scored 97.2%, nearly a 10% improvement. The FTDC plan improved PTV coverage and OAR sparing, most notably for the ribs and chestwall.

Table 3 shows the mean, standard deviation, and range values of DCE for all structures across the 50‐patient cohort that were optimized with FTDC and MRDC. Using the DVH metrics listed in Table 2, statistical analysis was performed in determining DCE_FTDC_ and DCE_MRDC_ for targets and thoracic OARs. Table 3 shows FTDC having improved agreement with AXB in terms of PTV coverage, as PTV D95% DCE_FTDC_ averaged less than 1%, as compared to an average DCE_MRDC_ of −2.5%. Paired *t*‐tests showed that FTDC significantly reduced DCE for both PTV D95% and D99% when compared to MRDC (*p*‐values less than 0.05 for both PTV D95% and D99%). The average DCE_FTDC_ for both PTV D95% and D99% were both within 1.5%, showing improvements in optimization dose calculation provided by FTDC for lung SBRT cases. PTV D99% DCE_MRDC_ was negative, meaning that MRDC overestimated PTV coverage. Of the 50 patients on the study, only 4 had a PTV D95% with DCE_FTDC_ greater than 2% or less than −2%, as compared to 22 cases that were optimized with MRDC. As for PTV D99%, both FTDC and MRDC exhibited noticeable statistical variation, with 23 and 27 plans, respectively, yielding a DCE outside of the ± 2% window; expanding that window to ± 5%, only one FTDC plan fell into this category, as compared to 15 plans in the MRDC cohort.

**TABLE 3:** DCEs for clinical lung SBRT dose constraints.

DCEs for PTV Goals and OAR Constraints						
Structure	DVH Metric	DCE_FTDC_		DCE_MRDC_		Paired *t*‐test
		Mean	Range	Mean	Range	*p‐*value
PTV	D95%	0.8 ± 0.9%	[−0.7%, 4.1%]	−2.5 ± 3.2%	[−11.4%, 1.8%]	**<0.05** ** <0.05**
	D99%	1.4 ± 1.7%	[−3.2%, 5.2%]	−2.6 ± 4.2%	[−16.6%, 2.8%]	
Chestwall	D30cc	−0.5 ± 0.4%	[−1.6%, 0.2%]	−1.1 ± 1.7%	[−7.7%, 1.4%]	>0.05
Esophagus	D5cc	−0.8 ± 2.7%	[−8.2%, 5.9%]	−2.1 ± 5.2%	[−18.9%, 5.0%]	>0.05
	D0.03cc	−0.9 ± 1.5%	[−7.4%, 1.1%]	−2.6 ± 3.3%	[−11.5%, 4.4%]	>0.05
GreatVes	D10cc	−0.6 ± 1.2%	[−3.8%, 4.8%]	−1.5 ± 2.2%	[−7.4%, 2.7%]	**<0.05**
	D0.03cc	−1.3 ± 2.1%	[−7.1%, 4.9%]	−2.8 ± 3.4%	[−12.1%, 3.6%]	**<0.05**
Heart	D15cc	−1.0 ± 1.9%	[−7.1%, 3.8%]	−1.1 ± 2.5%	[−7.5%, 7.5%]	>0.05
	D0.03cc	−2.1 ± 2.7%	[−10.5%, 0.9%]	−1.9 ± 3.5%	[−11.5%, 8.1%]	>0.05
Lung‐GTV	V1350cGy	−0.5 ± 1.1%	[−3.1%, 1.5%]	−3.2 ± 3.1%	[−13.3%, 3.2%]	**<0.05**
Ribs	D5cc	−0.6 ± 1.2%	[−4.0%, 4.5%]	−1.3 ± 2.3%	[−9.0%, 2.8%]	>0.05
	D0.03cc	−1.6 ± 1.7%	[−6.9%, 1.3%]	−1.1 ± 2.7%	[−9.5%, 5.1%]	**<0.05**
SpinalCord	D1.2cc	−0.8 ± 1.6%	[−6.8%, 2.6%]	−1.9 ± 4.2%	[−14.4%, 13.0%]	>0.05
	D0.35cc	−1.1 ± 2.2%	[−13.1%, 1.2%]	−2.0 ± 4.2%	[−17.8%, 13.9%]	>0.05
	D0.03cc	−1.8 ± 4.0%	[−25.9%, 4.9%]	−2.6 ± 5.3%	[−29.6%, 9.5%]	>0.05
Trachea	D5cc	−0.6 ± 1.2%	[−3.6%, 1.9%]	−3.1 ± 5.0%	[−13.7%, 4.3%]	**<0.05**
	D0.03cc	−1.1 ± 1.6%	[−4.5%, 3.1%]	−3.3 ± 4.7%	[−16.7%, 3.3%]	**<0.05**

*Note*: Mean and range values were presented for all structures across the patient cohort. Paired *t*‐test compare DCEFTDC versus DCEMRDC, with significance (p‐value < 0.05) shown in bold font.

In most scenarios, the DCE for thoracic OARs was negative for both FTDC and MRDC, with FTDC again exhibiting less statistical variation than MRDC for most OARs. Notably, Lungs‐GTV V1350cGy DCE was improved significantly by switching from MRDC to FTDC (*p*‐value < 0.05) for dose calculation, as FTDC (−0.5% ± 1.1%) clearly mirrored AXB better than MRDC (−3.2% ± 3.1%). Similarly, spinal cord, chestwall, and esophagus, the three OARs that were present within all fields, showed less statistical variation for plans optimized with FTDC than MRDC. Some OARs showed larger statistical variations for DCE DVH metrics, as compared to the PTV goals. Heart and trachea exhibited the greatest ranges and statistical variation, as seen in Table 3; however, these include plans with beams that did not enter or exit through these structures, and both MRDC and FTDC tended to underpredict scatter dose to these structures at large distances from isocenter; subsequently, paired *t*‐tests yielded *p*‐values greater than 0.05 for heart and other mediastinal OAR metrics.

### 3.2 Impact of target locations on DCE

**FIGURE 2:**
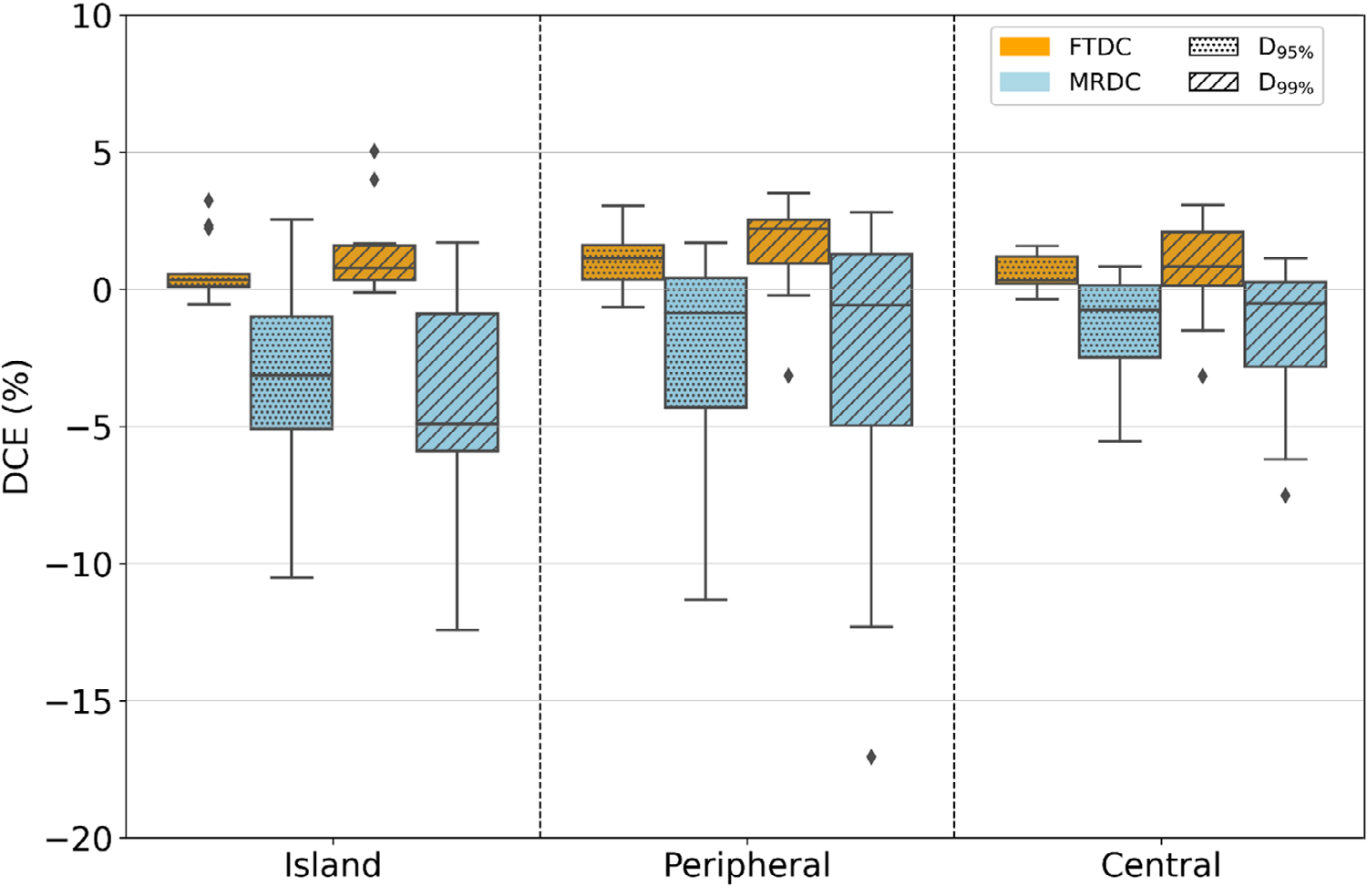
A box‐and‐whisker plot of PTV D95% and D99% DCEs by PTV location for plans utilizing FTDC and MRDC as the dose calculation algorithm during optimization. Clinical goals and constraints were attached to each plan to yield DVH metrics at the end of optimization and after final dose calculation. DCE, dose calculation error; DVH, dose volume histogram; FTDC, Fourier transform dose calculation; MRDC, multi‐resolution dose calculation.

As target location could impact PTV coverage, it is important to assess DCE for both algorithms in different locations across the lungs. Plans were split into three locations: central, peripheral and island. There were noticeable trends in DCE_FTDC_ and DCE_MRDC_ across these locations, as seen in Figure 2. While central lesions located near the mediastinum exhibited the smallest variations in PTV D95% DCE_MRDC_, averaging –1.4% ± 1.9%, DCE_MRDC_ worsened for peripheral (–2.2% ± 3.6%) and island (–3.1% ± 3.0%). FTDC improved PTV D95% DCE for all three sublocations, averaging 0.6% ± 0.6%, 1.1% ± 1.0%, and 0.6% ± 0.9% for central, peripheral, and island lesions, respectively. DCEs for peripheral PTVs (bordering chestwall and ribs) and island (surrounded by low density lung tissue in all directions) were greater, on average, for MRDC. FTDC compared favorably to AXB for peripheral and island PTV D95% and D99%, suggesting that surrounding tissue heterogeneity had less impact on FTDC calculation in the optimizer than MRDC. FTDC reduced PTV DCEs significantly for all three tumor locations (*p*‐value < 0.05).

**FIGURE 3:**
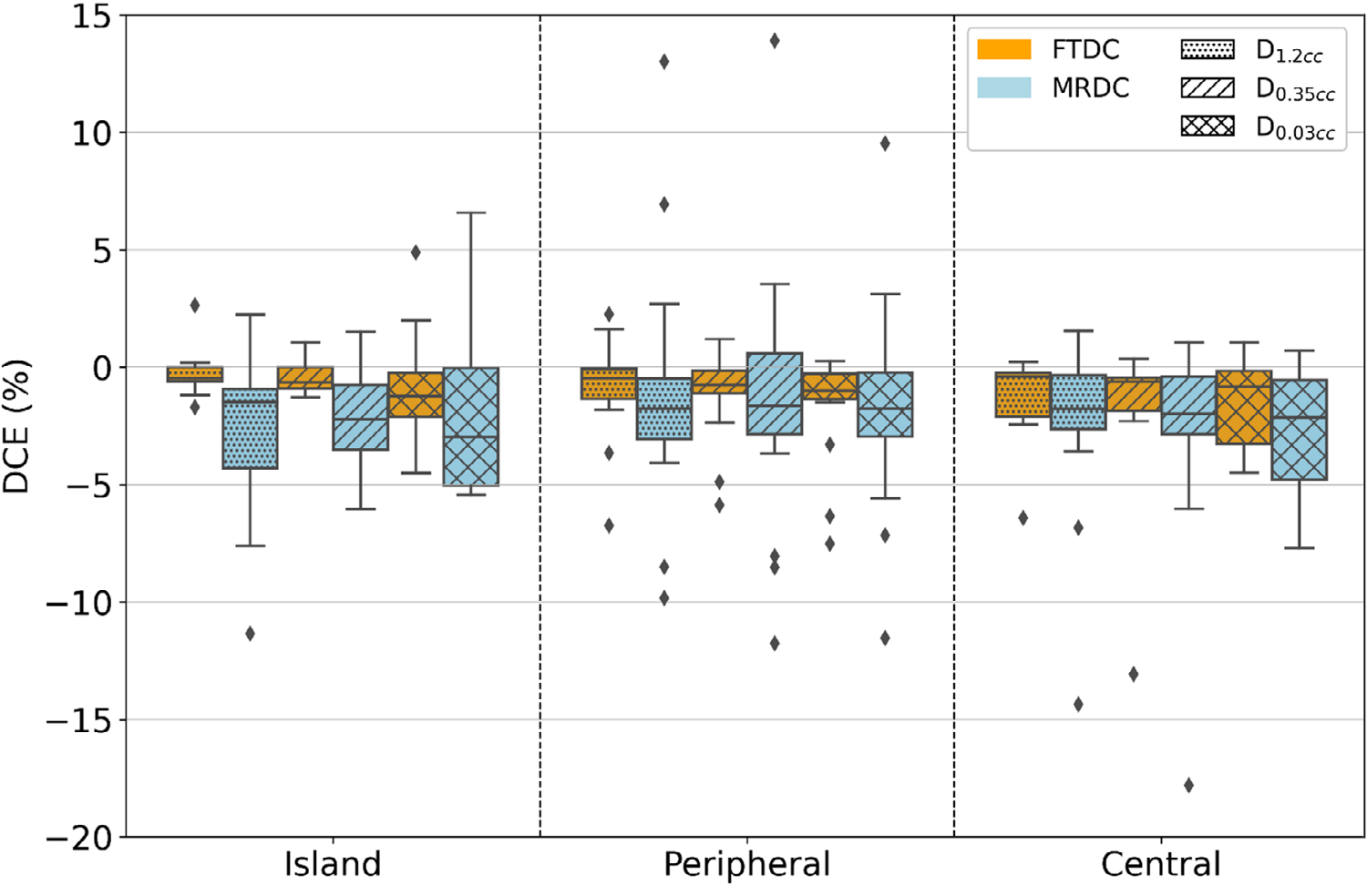
Box‐and‐whisker plots showing DCE for both FTDC and MRDC algorithms for all spinal cord metrics by tumor location. DCE, dose calculation error; FTDC, Fourier transform dose calculation; MRDC, multi‐resolution dose calculation.

Similar to PTV DCEs, there was a dependence on isocenter location on DCE for OARs. Across the different locations and OARs, it was apparent that DCE_FTDC_ was not greatly impacted by isocenter location, as compared to MRDC, which exhibited greater range, variability, and number of outliers. Plans with tumors located in the peripheral lung tended to show greater number of outlier statistics for central OARs, including spinal cord, esophagus, great vessels, trachea, and heart. Figure 3 shows DCE statistics for spinal cord broken down by tumor location. For peripheral cases, the distance from the lesion (i.e., isocenter) to the spinal canal was greater than that for most island and all centrally located lesions. Distance likely impacted dose calculation in the optimizer, as evidenced by the increased number of outliers and larger spread of DCE values for peripherally located lesions. Spinal cord also exhibited the largest range of DCEs, compared to the other OARs. One of the major differences in the spinal cord, as compared to the other OARs, is that this OAR is encased by vertebral bodies, with the bony anatomy possibly affecting the accurate dose calculation within the canal. For the other centrally located OARs, the range and variability of DCEs were again affected by tumor location, but not to as large of a degree as the spinal cord. Esophagus, great vessels, heart, and trachea D0.03cc DCE values followed a similar pattern. As for the peripherally located ribs and chestwall OARs, location seemed to have a similar effect on DCEs. Plans with centrally located lesions tended to have larger ranges and variability for OARs in terms of DCE_MRDC_. For most centrally located OARs, the percentage of arclength in which they fall within the beam path is minimal, especially for peripherally located tumors. Structures like esophagus and trachea tended to have larger ranges due to small overlap with arclengths and distance from the target, whereas ribs and chestwall, which had greater proximity to peripheral and central targets, exhibited less statistical variation, especially for FTDC plans.

**FIGURE 4:**
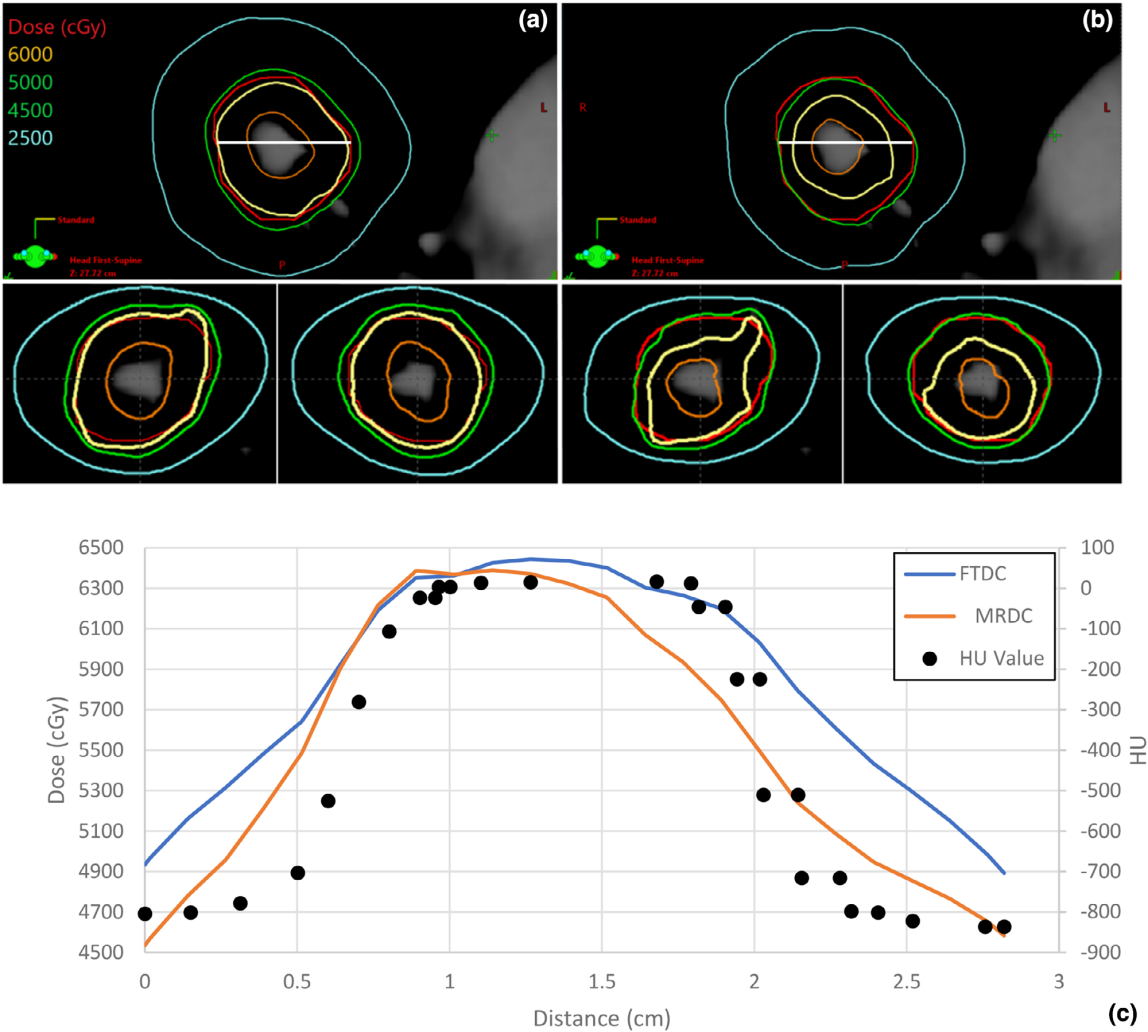
(a)–(c) Three‐views of patient 11 planned with FTDC (a) and MRDC (b). Horizontal dose and HU profiles are drawn through an island PTV. FTDC (blue) shows adequate PTV coverage (D95% of 4918cGy), as compared to MRDC (orange, 4502cGy), despite having very similar D95% values during the final step of plan optimization. PTV coverage dips with drops in HU along the dose profiles (white line drawn in 3a) for the plans using MRDC in PO. FTDC, Fourier transform dose calculation; MRDC, multi‐resolution dose calculation; PO, photon optimizer.

As a representative case, Patient 11, presenting with an island lesion with a PTV volume of 12.6 cc that was also largely low density (mean HU of −649, 96.8% of pixels have HU less than −100), epitomized the difference in optimizer dose calculation algorithms. FTDC and MRDC yielded PTV D95% of 4907 and 4934 cGy, respectively, at the final step in the optimizer; however, the results varied greatly at final dose calculation with AXB, as PTV D95% coverages of 4918 cGy and 4502 cGy were achieved for FTDC and MRDC plans, respectively. Whereas the PTV D95% DCE_FTDC_ was −0.23%, the DCE_MRDC_ was −8.8%. As seen in Figure 4a,b, the original isodose distributions show the improved coverage for FTDC (a) as compared to MRDC (b) for a small island lesion measuring 12.6 cc in volume. To further investigate the dose distribution, dose profiles were extracted from all three plans with a line profile drawn horizontally through the PTV. Figure 4c shows the plan optimized with MRDC falls short of the prescribed dose across the lateral line profile, especially as the HU values drop outside of the tumor on the average scan. FTDC, enhanced with TERMA scaling, not only led to greater synergy between dose calculated in the optimizer and AXB but can also improve planning efficiency.^14^ Multiple iterations of plan optimization would likely be needed to improve the coverage for the MRDC‐optimized plan, while a small normalization for the FTDC plan (±1%) could yield desired coverage without compromising plan quality or OAR constraints.

### 3.3 Impact of optimization and delivery strategies on DCE

The effect of optimization and delivery strategies on PTV D95% and D99% DCEs was examined further in Table 4. The magnitude of DCEs for PTVs showed greater dependence on optimization dose grid resolution than use of manual or KBP optimization. When enabling fine dose grid size in the optimizer, FTDC plans showed improved D95% and D99% DCEs, on average, compared to MRDC (0.1% ± 0.6% and 0.8% ± 1.1% vs. −3.4% ± 2.7% and −3.6% ± 3.1%). Plans using manual optimization tended to have less statistical variation, in terms of PTV D95% and D99% DCEs, than KBP plans for both FTDC and MRDC, suggesting that KBP did not greatly impact dose calculation accuracy between final and optimization dose calculation. Enabling fine grid resolution in the optimizer reduced the mean and standard deviation for DCE_FTDC_; the gap between DCE values was larger between FTDC and MRDC plans when fine resolution was utilized as compared to the default setting of 2.5 mm. DCE was also reduced when utilizing FTDC and HD‐MLCs; the combination of FTDC and HD‐MLC yielded the smallest mean and range for DCE values in terms of calculation algorithm and MLC type. Of note, paired t‐tests showed significance for each optimization and delivery strategy (*p*‐value < 0.05), showing that FTDC improved PTV DCEs for every scenario.

**TABLE 4:** PTV DCE values based on the use of KBP, structure resolution in PO, and MLC type.

DCE for FTDC and MRDC by Optimization Techniques and MLC Type						
		DCE_FTDC_		DCE_MRDC_		
Optimization Technique/Resolution/MLC Type	DVH Metric	Mean	Range	Mean	Range	Paired *t*‐Test *p*‐value
KBP	D95%	1.0 ± 1.0%	[−0.7%, 3.2%]	−2.6 ± 3.9%	[−11.3%, 2.5%]	**<0.05**
	D99%	1.3 ± 1.8%	[−3.2%, 5.0%]	−3.4 ± 5.3%	[−17.0%, 2.8%]	**<0.05**
Manual	D95%	0.4 ± 0.5%	[−0.4%, 1.6%]	−2.2 ± 2.4%	[−9.6%, 0.5%]	**<0.05**
	D99%	1.3 ± 0.9%	[−0.2%, 3.5%]	−2.2 ± 2.7%	[−9.9%, 1.3%]	**<0.05**
Fine Res (1.25 mm)	D95%	0.2 ± 0.6%	[−0.6%, 2.3%]	−3.6 ± 3.0%	[−10.5%, 0.6%]	**<0.05**
	D99%	0.8 ± 1.0%	[−0.2%, 4.0%]	−4.2 ± 3.8%	[−12.4%, 1.7%]	**<0.05**
Default Res (2.5 mm)	D95%	1.1 ± 0.8%	[−0.7%, 3.2%]	−1.9 ± 3.5%	[−11.3%, 2.5%]	**<0.05** **<0.05**
	D99%	1.6 ± 1.7%	[−3.2%, 5.0%]	−2.3 ± 4.8%	[−17.0%, 2.8%]	
HD‐MLC	D95%	0.6 ± 0.7%	[−0.7%, 2.3%]	−2.7 ± 3.4%	[−11.3%, 0.8%]	**<0.05**
	D99%	1.2 ± 1.5%	[−3.2%, 4.0%]	−3.1 ± 4.5%	[−17.0%, 2.6%]	**<0.05**
Millenium MLC	D95%	1.1 ± 1.1%	[−0.6%, 3.2%]	−1.9 ± 3.3%	[−9.6%, 2.5%]	**<0.05**
	D99%	1.5 ± 1.6%	[−1.5%, 5.0%]	−2.7 ± 4.6%	[−12.3%, 2.8%]	**<0.05**

*Note*: Paired *t*‐test compares DCE_FTDC_ versus DCE_MRDC_ for PTV D95% and D99% achieved by each optimization technique, resolution grid size, and MLC type; all *t*‐tests show significance (*p*‐value < 0.05).

### 3.4 Planning failure rate

**FIGURE 5:**
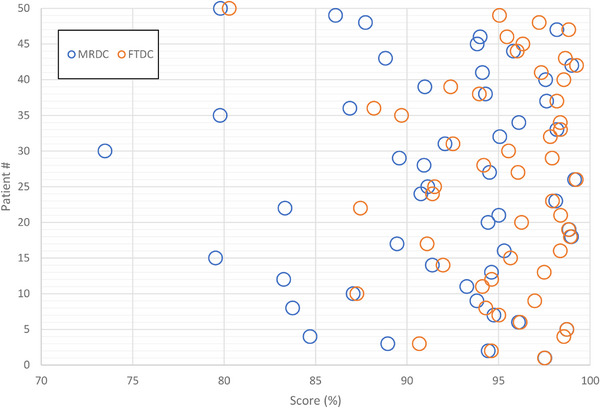
Barbell plots of dosimetric scorecard results for MRDC (blue) and FTDC (orange) plans for each patient. FTDC, Fourier transform dose calculation; MRDC, multi‐resolution dose calculation.

PlanScoreCard was utilized to assess plan quality for all plans in the study. Scores were out of 100%. Figure 5 highlights the scores for both MRDC and FTDC plans for the entire cohort of patients. FTDC had a PFR of 10% (5 out of 50) versus MRDC's 32% (16 out of 50). The subsequent re‐optimization rate resulted from a plan normalization of 3% or greater was 4% for FTDC compared to MRDC's 38%.

### 3.5 Optimization efficiency

MRDC optimization times for all patients averaged 15.8 ± 13.7 min. FTDC, boosted with GPU assisted computation, exhibited the shortest optimization duration, with an average of 4.0 ± 2.8 min across the entire patient cohort, a time savings of nearly 75% from MRDC. For comparison, optimizing FTDC plans (FTDCc) with CPUs enabled, rather than GPUs, averaged 10.1 ± 7.4 min, still faster on average than MRDC, as seen in Table 5. Paired *t*‐tests show that FTDC, regardless of GPU assistance, significantly improved plan optimization time (*p*‐values < 0.05).

**TABLE 5:** Optimization times (in minutes) for all plans.

Optimization time (in minutes) for FTDC (CPU and GPU enabled) and MRDC				
	Optimizer Settings	FTDC (CPU)	FTDC (GPU)	MRDC
Total	All plans	10.1 ± 7.4	4.0 ± 2.8	15.8 ± 13.7
Structure resolution	2.5 cm	6.2 ± 3.0	2.8 ± 1.0	10.2 ± 6.8
	1.25 cm	17.5 ± 7.5	6.6 ± 3.5	27.4 ± 17.0
KBP use	KBP	10.1 ± 8.0	3.9 ± 3.2	18.4 ± 17.6
	Manual	9.6 ± 6.5	4.1 ± 2.3	13.4 ± 7.9
Combined effect of Structure Resolution and KBP Use	1.25 cm, Manual	15.9 ± 5.3	5.8 ± 2.1	20.0 ± 5.4
	1.25 cm, KBP	24.2 ± 9.4	8.4 ± 5.7	48.2 ± 22.3
	2.5 cm, Manual	4.5 ± 0.9	2.4 ± 0.6	6.8 ± 2.1
	2.5 cm, KBP	7.3 ± 3.3	3.0 ± 1.2	12.4 ± 7.7

*Note*: FTDC with CPU based calculation was included for comparison with FTDC with GPUs enabled. Optimization time was further broken down into plans that used normal (2.5 mm) and high (1.25 mm) structure resolution and KBP and manual optimization methods, as well as all four combinations.

Abbreviations: FTDC, Fourier transform dose calculation; GPU, graphic processing unit; KBP, knowledge‐based planning; MRDC, multi‐resolution dose calculation.

Further investigation into optimization time involved the implementation of KBP modeling and high‐resolution dose calculation during the inverse planning process. We have seen clinically that both KBP modeling and high‐resolution settings slow the optimizer, as compared to using manual optimization and normal resolution. Plans using high resolution dose calculation tended to require twice as much time to reach the end of MR Level 4 in PO, as seen in Table 5. The use of KBP models did not have a significant influence on optimization time for FTDC but plans using MRDC experienced longer optimization times by 5 min on average (18.4 ± 17.6 min vs. 13.4 ± 10.7 min). Utilizing both high resolution and KBP did lead to longer optimization times for all algorithms. Switching from MRDC to FTDC could achieve a time saving of up to 83%, or nearly 40 min, when both KBP and high‐resolution settings are utilized in the optimizer; note that all optimization times assume that the planner is not adjusting objectives and priorities and is allowing the optimizer to converge during each step of the multi‐resolution optimization process.

### 3.6 Delivery efficiency

FTDC plans exhibited an improvement in delivery efficiency, as the total MU for FTDC plans (average: 2817 ± 409 MU) show more than a 15% improvement from MRDC (average: 3341 ± 489 MU). Similarly, FTDC reduced overall plan complexity by nearly 30% (0.16 ± 0.04) from the original MRDC plans (0.23 ± 0.05).

## 4 DISCUSSION

### 4.1 Dose calculation accuracy

For patients receiving lung SBRT, achieving optimal treatment plans with the most accurate dose calculation and highest optimization efficiency is of utmost importance. With its increasing number of clinical applications, SBRT is often relied on to treat lung lesions adjacent to critical normal structures (i.e., pericardium, chest wall, or bronchus), treatment of multiple lung lesions either simultaneously or sequentially, and in cases of re‐irradiation. These types of cases underscore the importance of accurate dose calculation, as a difference between the expected and delivered dose of a few percent could potentially have important clinical implications.

It is possible that superior plan efficiency can be achieved with highly accurate dose calculation algorithms during and after plan optimization. In this work, we examined DCEs and PFRs associated with the dose calculation algorithms available in PO18. Shifting from MRDC to FTDC during plan optimization reduced the range of DCEs for PTVs and OARs alike; DCEs for PTV D95% ranged from −11.4% to 1.8% with MRDC, while FTDC reduced that range from −0.7% to 4.1%. In terms of plan quality, FTDC reduced the PFR from MRDC's 38% to 4%, thus greatly improving the planning efficiency.

With lung SBRT planning, heterogeneous tissue densities and small field sizes complicate the accuracy of dose calculation algorithms. Whilst AXB and MC have closed the gap between calculated and measured dose distributions,^22^, ^23^, ^24^, ^25^ simplified dose calculation algorithms used during VMAT optimization have tended to lag and shown disparities in the form of DCEs and optimization convergence errors that were reported in other studies.^7^, ^9^ With the arrival of Eclipse v18, the chasm between dose calculation within PO and AXB has been shortened, even for complex cases such as lung SBRT where low‐density regions and small field sizes could lead to greater sources of error. With most lung PTVs containing low density lung tissue within the volume, it is imperative for the dose calculation within the optimizer to model dose deposition in these regions properly. FTDC greatly reduced the lower end of the range, meaning that dose calculated to the PTV during optimization has improved and requires little to no plan normalization after AXB calculation to achieve desired PTV coverage. However, the upper range was over 2% higher. In revisiting these cases, we noticed that the planner had asked for excessive PTV coverage in the optimizer. With FTDC, planners can relax lower limits and priorities for targets while knowing that final dose calculation will more closely mirror the dose distributions and DVH seen in PO18.

### 4.2 Sources of DCE

In this study, we aimed to identify sources of DCEs in lung SBRT plans in Eclipse. Dogan et al. listed potential sources of dose prediction and convergence errors, including improper fluence prediction, handling of tissue heterogeneities, and differences in beam modeling between optimization calculation and final dose calculation algorithms.^9^ Utilizing FTDC can limit some of these potential sources of error that can propagate through plan optimization and generation. First, FTDC is a convolution/superposition algorithm and should reduce some of the inherent errors attributed to pencil beam algorithms within plan optimization. MRDC is a more simplistic dose calculation algorithm, relying on pencil beam formalisms to boost efficiency. Jeraj et al. found systematic errors of 8% ± 3% for lung tumors when employing pencil beam algorithms in the optimizer, versus just −0.1% ± 2% when using a more accurate superposition algorithm.^10^ The larger systematic errors associated with pencil beam contributed to larger convergence errors for tumors and nearby OARs in lung, pelvis, and head and neck IMRT plans, suggesting that DCEs and OCEs could be reduced by using a more accurate dose calculation algorithm during both optimization and final dose calculation.

Similar to Dogan's work, we believe that one of the greatest sources of DCEs in dose calculation is the effect of tissue homogeneities.^9^ PTV D95% and D99% DCE_MRDC_ values were greatly impacted by location, as differences between dose in the optimizer and final dose calculation rose as the volume of PTV enveloped by low‐density lung tissue increased. Island lesions pose an issue due to lack of buildup and large percentage of lung volume comprising the PTV. Previous studies have shown discrepancies between Monte Carlo and Pencil Beam of up to 30%.^26^ Other studies have shown over‐prediction of PTV coverage was greater for island lesions than peripheral lesions when AAA was used for dose calculation, as compared to AXB.^25^ In our study, PTV D95% and D99% DCE_FTDC_ were not greatly impacted by location, which can partially be attributed to the TERMA scaling introduced in the updated FTDC algorithm.^16^

Another potential source of DCEs in this work is MLC modeling and its effect on fluence prediction and dose calculation. A new feature in Eclipse v18.0 is the Enhanced Leaf Model (ELM), which shows improved MLC modeling for Millenium, High Definition, and Halcyon MLC models. The ELM models attenuation through the drive screw and leaf tip more accurately and adopts a more divergent ray tracing method for leaf transmission.^28^ The photon calculation algorithms in Eclipse v18 employ ELM within Beam Configuration. In keeping all final dose calculations consistent in this work, all calculations were performed with AXB18. However, ELM was also attached to the PO18 algorithm utilized during optimization. For FTDC, ELM was accounted for during plan optimization, but PO15, which does not rely on ELM, was utilized in MRDC plans. The work from van Esch et al. showed the effects of ELM were much greater for MLC fields that were further off‐axis in the *X*‐direction; several MLC tests described in this work show smaller differences between the original MLC model and updated ELM model at isocenter.^28^ For all plans in this study, isocenter was aligned with the center of the PTV; keeping the isocenter at the center of the target for all cases was meant to reduce potential effects of ELM and retain the focus on dose calculation algorithms. Attempts to reduce modulation during optimization were utilized to prevent MLCs from blocking the PTV; aperture settings in PO were set to high or very high to reduce modulation. In aiming to reduce MLC modulation, we believed we could also limit the effects of ELM, as the leaf tip and drive screw modeling could also impact the differences seen between MRDC and FTDC. As Dogan et al. pointed out, inaccurate MLC modeling could contribute to DCEs.^9^ While we made attempts to minimize the effects of ELM, it is possible that the combination of improved MLC modeling and FTDC led to reduced DCEs for targets and OARs. The impact of ELM within PO is currently unknown, as, to our knowledge, this is the first work exploring PO18 in the clinical setting.

Our PTV DCEs were consistent to those in Dogan's study on head and neck IMRT.^9^ While our study focused on lung SBRT with VMAT, our study was structured in a similar manner and involved a treatment site that involved many tissue heterogeneities. However, our ranges of DCEs tended to be much higher than those in the head and neck region, especially for the case of spinal cord. It is possible that another source of DCEs introduced is calculation accuracy at greater depths along the arc path length. With lung tumors in various regions of the lung and arcs rotating partially around the patient, OARs can be at large distances away from isocenter. The effective distance of OARs from isocenter can potentially play a role in calculation accuracy, as some OARs were routinely over 10 cm away from targets throughout the arc paths, and in many cases, did not overlap with the arc path lengths. DCEs were also excessive for cases where the OAR was not in the arc paths altogether, suggesting scatter dose was not accurately predicted by either MRDC or FTDC in the optimizer.

### 4.3 Plan complexity

Plans optimized with FTDC saw a reduction in MUs as compared to MRDC‐optimized plans, with a reduction of 15% on average. Plan complexity scores were also reduced by 30%. Similar improvements have been noted in previous algorithm upgrades; Liu et al. noted that switching from PRO to PO for lung SBRT planning reduced total MUs by 30%.^11^ A reduction in MUs could potentially reduce out‐of‐field dose and secondary cancers.^29^ Reducing plan complexity can also decrease the impact of small field sizes, as well as the interplay effect for moving lung lesions.^16^, ^30^ According to Laakkonen et al., the new FTDC algorithm performs well for small field sizes down to 10 × 10 mm^2^ in heterogeneous phantoms; FTDC compared more favorably than AAA in capturing the depth dose curve for fields as small as 10 × 10 mm^2^ for 6MV photon fields.^16^ In a comparison of high and low complexity lung SBRT VMAT plans, Ge et al. noted that two and three arc low complexity plans were less susceptible to respiratory motion and had a higher delivery accuracy than their high complexity counterparts.^30^

### 4.4 Planning efficiency

FTDC, assisted by GPU, showed great improvement in plan optimization efficiency when compared to MRDC with CPU. With the ability to utilize GPUs during the optimization process, FTDC was nearly four times faster than MRDC in this study. Plan optimization that is relying on KBP modeling and calculating on a high‐resolution dose grid would greatly benefit from the use of FTDC. With the need to generate complex plans with a short turnaround time in busy clinics, FTDC could improve treatment planning efficiency for a wide range of cases and could benefit departments with active off‐line adaptive radiotherapy programs.

### 4.5 Plan quality

It's worth revisiting the idea from two decades ago that better treatment plans can be achieved when more accurate dose calculation algorithms are utilized throughout the treatment planning process, from the inverse optimization through final dose calculation. In our work, dosimetric scorecard analysis showed clear improvements in plan quality when FTDC was utilized in the optimizer. Dosimetric scorecards can provide detailed feedback on treatment plans; the lung SBRT scorecard we crafted analyzed plans based on our department standards, akin to our dosimetric goals and constraints, with added emphasis on PTV coverage and OAR sparing. FTDC plans significantly outscored MRDC plans (*p‐*value < 0.01). Only 5 original FTDC plans scored less than 90% (PFR of 10%) whereas 16 MRDC plans scored lower than 90% (PFR of 32%). FTDC yielded 1 case with a score less than 85%, whereas 8 (16%) of MRDC cases yielded scores less than 85%; the one FTDC outlier was due to a large tumor volume, thus reducing scores for intermediate dose falloff and surrounding OARs. In our department, plans scoring below 90% would be scrutinized clinically, as either target coverage was not met, or one or more OARs did not meet clinical tolerances. In some cases, simply normalizing the plan could improve target coverage and lead to an increase in plan score. Plan normalization presents inherent risks of increasing hot spots within targets and OARs; it is not always certain that a simple normalization would improve plan scores. Large plan normalizations could force planners to reoptimize, thus reducing plan efficiency and contributing to the high re‐optimization rate for plans using MRDC during plan optimization.

## 5 CONCLUSION

The launch of Eclipse TPS v18 has brought forth improvements in VMAT optimization, namely in dose calculation accuracy and optimization efficiency, with the arrival of FTDC for clinical use. In our clinical study, FTDC shows closer correlation in dose distributions with AXB than its predecessor, MRDC. Not only was dose calculation accuracy improved for lung SBRT plans, which inherently brings the issue of heterogeneity and small field size dosimetry into play, but also plan optimization efficiency was great enhanced. FTDC gives clinicians the ability to achieve the desired dose distribution they are pushing for during plan optimization, which could coincide with improved treatment plans and increased planning efficiency.

## AUTHOR CONTRIBUTIONS

Kenny Guida—lead author, treatment planning, data collection, manuscript review and preparation. Chaoqiong Ma—data analysis, data preparation, and manuscript review. Joy Patel—treatment planning and manuscript review. Krishna Reddy—manuscript review and preparation. H Harold Li—senior author, manuscript review and preparation

## CONFLICT OF INTEREST STATEMENT

Dr. Guida reports honoraria from Varian Medical Systems outside the submitted work. Dr. Reddy reports honoraria from Varian Medical Systems outside the submitted work.

## Figures and Tables

**FIGURE A1:**
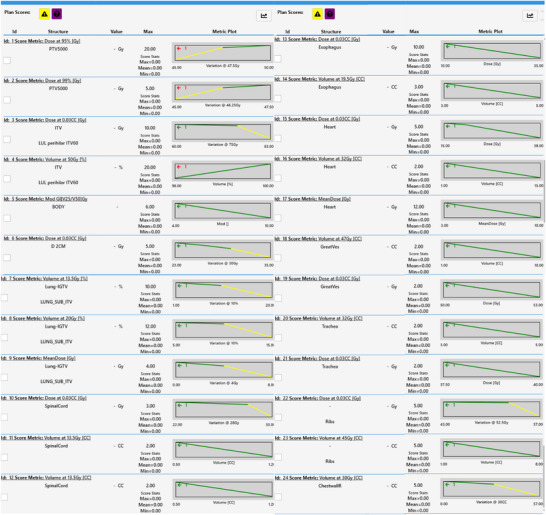
Dosimetric Scorecard generated for assessing MRDC and FTDC plans. Scorecards were utilized to qualitatively assess plans and rank the plans yielded by optimizing with both calculation algorithms. FTDC, Fourier transform dose calculation; MRDC, multi‐resolution dose calculation.

## APPENDIX A

A dosimetric scorecard was utilized to evaluate all plans in this work. The Plan Scorecard was launched via ESAPI script (PlanScoreCard) and applied to MRDC and FTDC plans, both normalized and unnormalized, to generate a score. A total of 154 points could be achieved. 100% is the maximum score achievable. All scores were given as a percentage of the maximum achievable points. Clinically viable plans tend to have scores 90% or greater, as planners should strive to achieve a balance between target coverage, dose falloff, and OAR sparing. The figure below (Figure A1) shows how the plan scorecard was configured to score each plan.
